# Neurosurgical simulator for training aneurysm microsurgery—a user suitability study involving neurosurgeons and residents

**DOI:** 10.1007/s00701-020-04522-3

**Published:** 2020-08-11

**Authors:** Fredrick Johnson Joseph, Stefan Weber, Andreas Raabe, David Bervini

**Affiliations:** 1 ARTORG Center for Biomedical Engineering Research, University of Bern, Bern, Switzerland; 2 Department of Neurosurgery, Bern University Hospital and University of Bern, 3010 Bern, Switzerland

**Keywords:** Resident training, Surgical simulation, Surgical education, Microsurgery, Intracranial aneurysm, Neurosurgery

## Abstract

**Background:**

Due to its complexity and to existing treatment alternatives, exposure to intracranial aneurysm microsurgery at the time of neurosurgical residency is limited. The current state of the art includes training methods like assisting in surgeries, operating under supervision, and video training. These approaches are labor-intensive and difficult to fit into a timetable limited by the new work regulations. Existing virtual reality (VR)–based training modules lack patient-specific exercises and haptic properties and are thus inferior to hands-on training sessions and exposure to real surgical procedures.

**Materials and methods:**

We developed a physical simulator able to reproduce the experience of clipping an intracranial aneurysm based on a patient-specific 3D-printed model of the skull, brain, and arteries. The simulator is made of materials that not only imitate tissue properties including arterial wall patency, thickness, and elasticity but also able to recreate a pulsatile blood flow. A sample group of 25 neurosurgeons and residents (*n* = 16: early residency with less than 4 years of neurosurgical exposure; *n* = 9: late residency and board-certified neurosurgeons, 4–15 years of neurosurgical exposure) took part to the study. Participants evaluated the simulator and were asked to answer questions about surgical simulation anatomy, realism, haptics, tactility, and general usage, scored on a 5-point Likert scale. In order to evaluate the feasibility of a future validation study on the role of the simulator in neurosurgical postgraduate training, an expert neurosurgeon assessed participants’ clipping performance and a comparison between groups was done.

**Results:**

The proposed simulator is reliable and potentially useful for training neurosurgical residents and board-certified neurosurgeons. A large majority of participants (84%) found it a better alternative than conventional neurosurgical training methods.

**Conclusion:**

The integration of a new surgical simulator including blood circulation and pulsatility should be considered as part of the future armamentarium of postgraduate education aimed to ensure high training standards for current and future generations of neurosurgeons involved in intracranial aneurysm surgery.

## Introduction

Because of its complexity and the alternative treatments available, neurosurgery trainees today have limited exposure to microsurgery of intracranial aneurysms [1–3]. Exposure to aneurysm clipping usually occurs during the advanced phase of a residency program or, for a select group, during a dedicated fellowship [4, 5]. The current state-of-the-art approaches are typically labor-intensive and difficult to fit into a time schedule depending on the work regulations. Other surgical disciplines have successfully implemented simulation tools and considered them as an effective training method [6, 7]. However, existing neurosurgical training models, like static simulators and virtual reality (VR) training, do not provide a realistic experience and are hard to use for limited and complex surgical anatomy [8–12]. Intracranial aneurysm microsurgery is also difficult to practice on ex vivo models (human or animal specimens) because vascular pulsatility cannot yet be realistically replicated [1, 13–15]. To successfully train neurosurgeons to operate under realistic conditions, a simulation tool should replicate the risk of aneurysm rupture, provide them with a realistic experience of microsurgical manipulation, and give them a thorough knowledge of the morphology of the brain and surrounding vascular structures [3, 11, 16].

3D aneurysm surgical simulators and physical models have been reported, but they lack blood-like flow time resolution and other crucial components like patency, dynamic behavior, haptics, tactility, and feedback for handling micro-surgical tools and clips [2, 17]. Existing models to date cannot expose trainees to the entire surgical workflow, which includes indocyanine-green video angiography (ICG) for inspecting aneurysm occlusion [18, 19]. Because the aneurysm wall and sac are very delicate, it is also difficult to mimic and simulate the sub-millimeter field [20, 21]. Today’s residents are more likely to learn through the conventional process, resulting in a global shortage of surgical exposure and making it difficult to train qualified cerebrovascular neurosurgeons [4].

Functional training simulators that replicate the required surgical anatomy and physiology could address this problem. In the last few years, manufacturing techniques have improved enough to produce true scale models of a patient-specific intracranial aneurysm, based on image data from real patients [22, 23]. We have developed a bench-top simulator that meets this need for realistic neurosurgical training models with blood flow and pulsatility, an additional dimension.

Our goal was to assess the realism of the simulation model and determine if it is feasible to use the simulator to train residents and neurosurgeons in intracranial aneurysm microsurgery.

## Material and methods

### Simulator and phantom

Image datasets from a patient with a left 14-mm middle cerebral artery (MCA) bifurcation aneurysm were used, including various imaging modalities (e.g., digital subtraction angiography [DSA], computer tomography [CT], and magnetic resonance images [MRI]) for thresholding-based segmentation with Amira 6.3 (Thermo Fischer Scientific, MA, USA). No additional special techniques were employed. True scale hard-tissue and soft-tissue portions of the required anatomy (skull, brain, intracranial arteries, and aneurysm) were 3D printed and produced according to standard additive manufacturing principles [24].

**Fig. 1:**
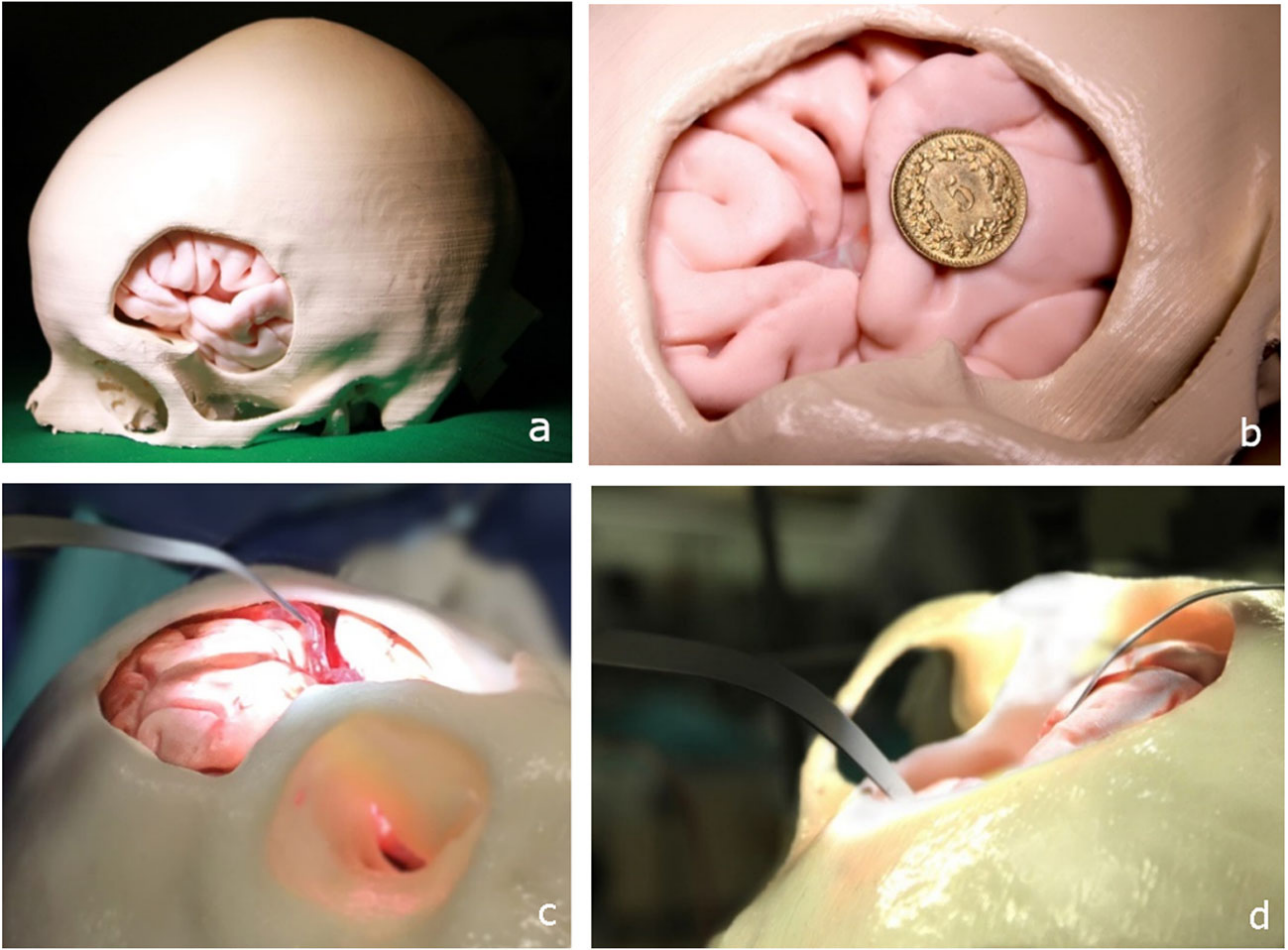
Representation of the model during the training study: **a** Patient-specific 3D-printed trephined skull with brain model. **b** MCA aneurysm model located in the left Sylvian fissure. **c** Pulsating blood vessel and access to the pathology of the model. **d** Brain retractor during manipulation by resident

We selected materials that replicate human mechanical and haptic properties [25, 26]. A standard left pterional craniotomy approach was made in the 3D-printed skull using Solidworks 2019 (Dassault Systems, France), as shown in Fig. 1a. The physical models, including the vascular anatomy, were positioned, assembled, and spatially oriented (Fig. 1b) using the image datasets and according to the patient’s anatomy [27]. The replica of the left distal internal carotid artery was connected to a custom-designed pulsatile pump able to reproduce a patient’s cardiac physiology (heart rate 50–110 BPM) and blood pressure (60–100/100–140 mmHg). A software application controlled the pumping unit to vary the heart rate and pressure, but we kept them constant for the purposes of this study (heart rate 70 BPM; blood pressure 80/120 mmHg). The continuous, pulsatile blood circulation was connected to a reservoir, closing the loop. The blood flow emulates haptic and optical behavior when mixed with ICG dye (VERDYE 5 mg/ml, Diagnostic Green GmbH, Germany).

The head model was placed and fixed with an adjustable 6-degrees of freedom arm to properly position the head (flexion/extension and rotation). The brain retractors were part of the simulator, but it was up to surgeons and participants to decide whether or not to use them. A neurosurgical microscope (OPMI® Pentero® 600, Carl Zeiss Surgical GmbH, Germany) was included in the bench-top training module.

### Study arrangements and participants

This study included 25 neurosurgery residents and board-certified neurosurgeons from several countries. Most participants had chosen cerebrovascular neurosurgery as their subspecialty focus (Table 1). To evaluate the quality of the training, we divided participants into two groups based on their surgical exposure and experience: group A included those in their early residency (< 4 years of full training); group B included those in their late residency (at least 4 years of full training) and board-certified neurosurgeons. Of the 9 participants in group B, 3 had over 4–15 years of neurosurgical experience, had clipped 10–150 aneurysms, and had more advanced neurosurgical experience than the other participants. The other 6 participants in group B were less experienced or had never performed neurovascular surgeries without supervision. Group A included 16 doctors in early residency, of mixed experience levels that varied from a few months to almost 4 years. All 16 participants in group A had assisted or viewed neurovascular procedures like aneurysm clipping but none had clipped an aneurysm (Table 1).

**Table 1:** Overview of participants and background

	No. of participants *n* = 25				
Years of neurosurgical experience	Group A: < 4 years Early residency	Group B: 4–15 years Late-residency and board-certified neurosurgeons			
No. of participants in each group	16	2	2	2	3
Background	Neurosurgery-resident	Functional neurosurgery	Pediatrics and tumor	Vascular neurosurgery	Vascular neurosurgery
No. of aneurysm clipped as the main surgeon	0	0			10–150

Participants were provided with a 2D DICOM image dataset of the patient model in order to anticipate the vascular anatomy and the clipping strategy. Five copies of the head replica were available and ready to switch into the simulator if the aneurysm ruptured during training.

### Study design

**Fig. 2:**
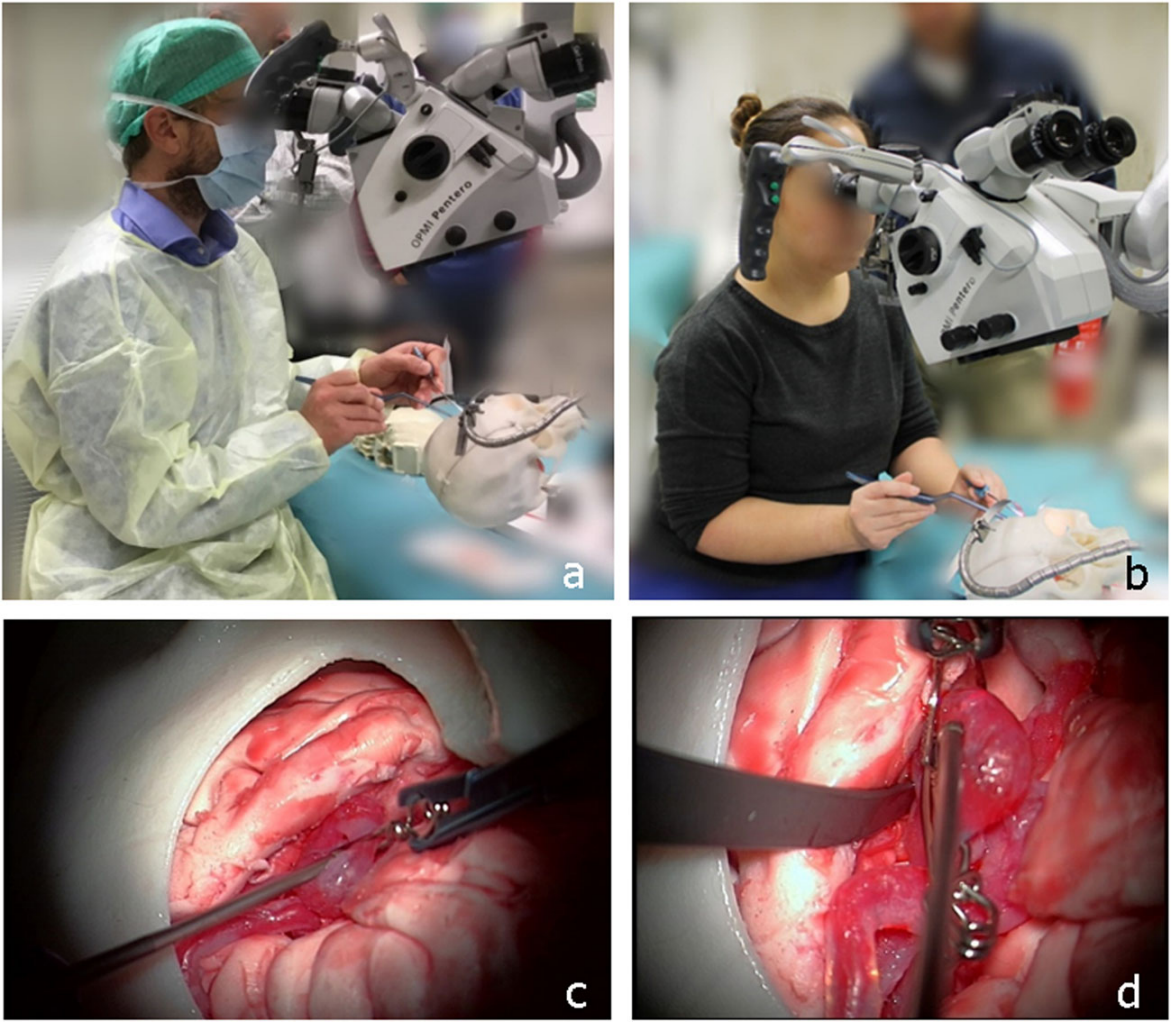
Pictorial representation from the simulator and study participation: **a** Expert neurovascular surgeon manipulating the aneurysm model in the simulator and trying to clip. **b** Young resident neurosurgeon clipping. **c** Attempt to clipping. **d** Exploration after clipping

State-of-the-art microsurgical instruments, including intracranial aneurysm clips, manipulators, and dissectors, were available for the training. Participants viewed the mimicking pathology through a surgical microscope (OPMI® Pentero® 600, Carl Zeiss Surgical GmbH, Germany). They manipulated the vessel structures before they chose the correct clip configuration (Fig. 2). Participants were given only one attempt to clip. Blood-like pulsatility through the model was held constant for all participants. The clipping procedure was recorded under the microscope and used by a blinded fully trained cerebrovascular neurosurgeon (DB) to assess the quality of each participant’s clipping after the training. The procedures were scored after reviewing ICG imaging (aneurysm obliteration: complete vs. incomplete) and assessing normal artery patency. Clipping results fell into one of three categories: (a) successful clipping (complete aneurysm occlusion, normal artery patency); (b) partial clipping (if clipping resulted in only partial aneurysm occlusion and normal artery patency); and (c) failed to clip/unsuccessful clipping ((1) could not attempt clipping, (2) aneurysm sac unsuccessfully occluded, (3) normal artery patency compromised, (4) aneurysm rupture). After a participant had clipped the aneurysm and marked the attempt as complete, she or he was asked to complete the study questionnaires (Table 2).

**Table 2:**
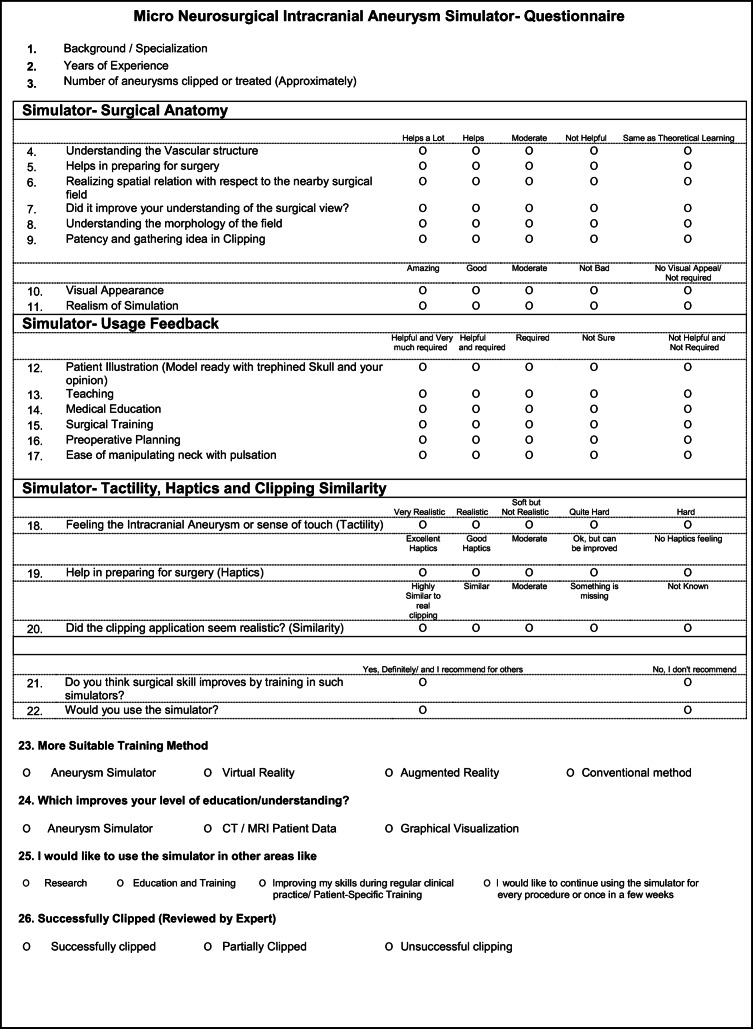
List of questionnaire

### Survey instrument development and administration

Attitudinal questions were formatted as 5-point Likert scales (Table 2). Some questions were multiple-choice questions to provide insight into the ease of use of this new training approach. Study data were collected and managed in REDCap 9.7.8 (Research Electronic Data Capture, Vanderbilt University) electronic data capture tools, hosted at ARTORG Center, Switzerland [28, 29].

### Statistical analysis and study outcomes

Survey responses were coded and double-checked for accuracy. Survey data was statistically analyzed with descriptive statistics for different endpoints with dedicated software (R Studio 1.2.5 Programming, 2020). The primary aim of the study was to determine if it is possible to use the simulator to train residents and neurosurgeons. We compared the clipping performance of each study group to find out if we could use the simulator to conduct a validation study that would measure the learning curve of residents as they train with the simulator.

## Results

### Surgical anatomy simulation

**Fig. 3:**
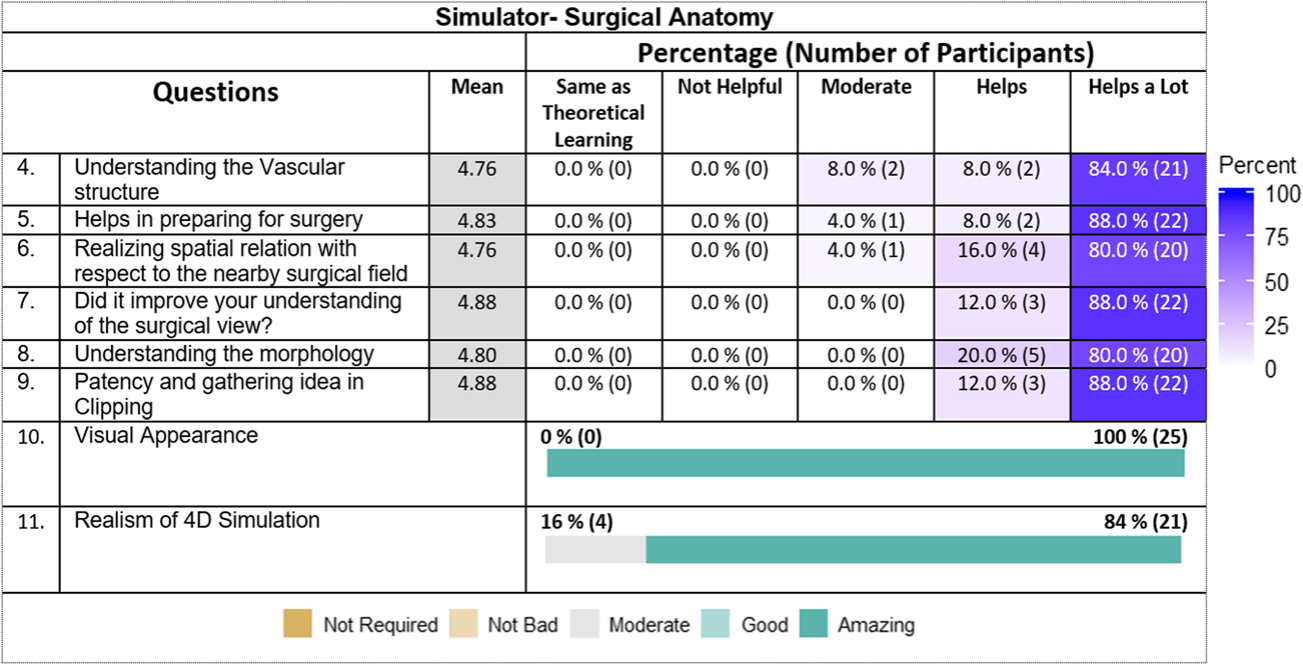
Likeability scoring-neurosurgical simulation anatomy

**Fig. 4:**
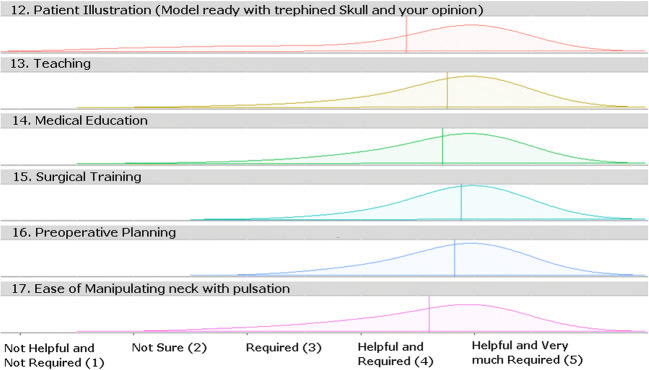
5 Likert scale score on the usefulness of the simulator about a different area of applications

Participants were asked eight questions about simulation anatomy to determine how closely the simulation models emulated reality and to compare the effectiveness of training with the simulator with that of the theoretical learning process. Many respondents scored the benefits of the model in a range differing from helpful to same as of theoretical learning. A large majority (84%; *n* = 21) found that the simulator improved their understanding of microvascular structures and anatomy far more than theoretical learning. A similar number (88%; *n* = 22) found that the simulator was of great help in preparing them for surgery. Most (80%; *n* = 20) found the simulator very useful for teaching them the neighboring anatomy and the same number (80%; *n* = 20) found that the simulator helped them learn the morphology of the surgical field so they could differentiate the surrounding anatomy from the pathology. Most participants (88%; *n* = 22) thought this novel training method improved their understanding of surgery and was more useful than theoretical learning for the understanding of flow patency during aneurysm clipping. Most participants (84%; *n* = 24) found the clipping simulation to be as realistic as surgery and all participants felt that the global visual appearance of the model was realistic. Differences in their responses to the simulation anatomy are shown in Fig. 3. Figure 4 shows the responses on general usability feedback and future use areas of the simulator.

### Haptics, tactile feedback, and clipping similarity

**Fig. 5:**
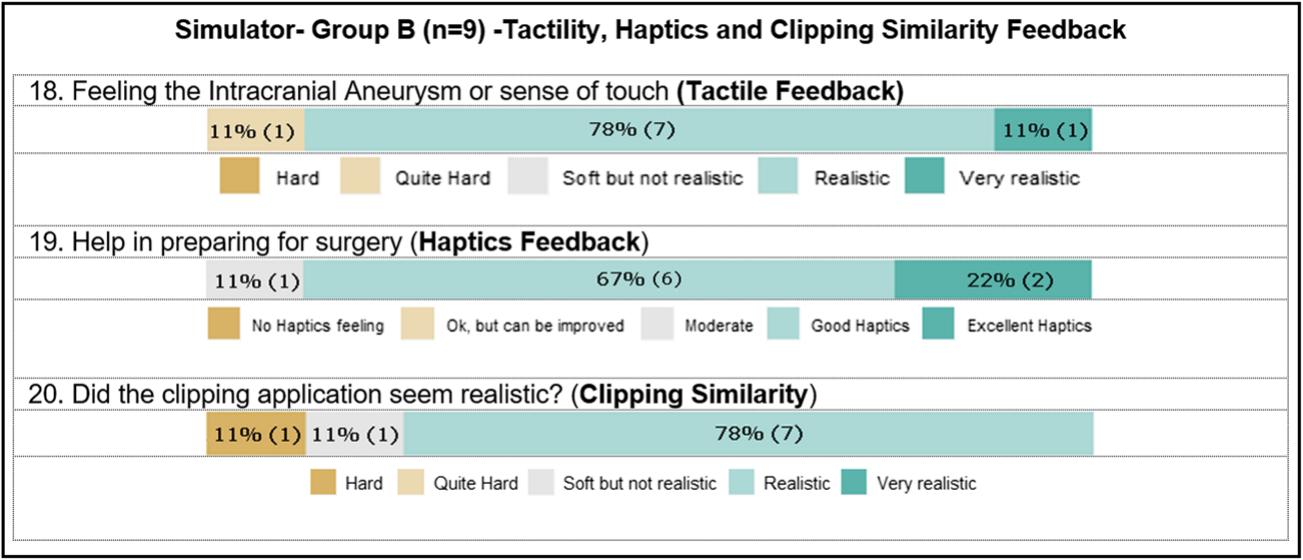
Likert user rating on the simulation training evaluating the tactile, haptics, and clipping similarity during the training process

Three questions on haptics, tactility, and clipping similarity were asked only of group B (late residency and board-certified neurosurgeons) in order to assess the unique features of the simulator. Almost all group B members (89%; *n* = 8/9) felt that the model’s tactility while clipping was realistic. The same number found the haptics realistic, and most (78%; *n* = 7) found that the simulation resembled real clipping surgery (Fig. 5).

### Status of clipping nature

**Fig. 6:**
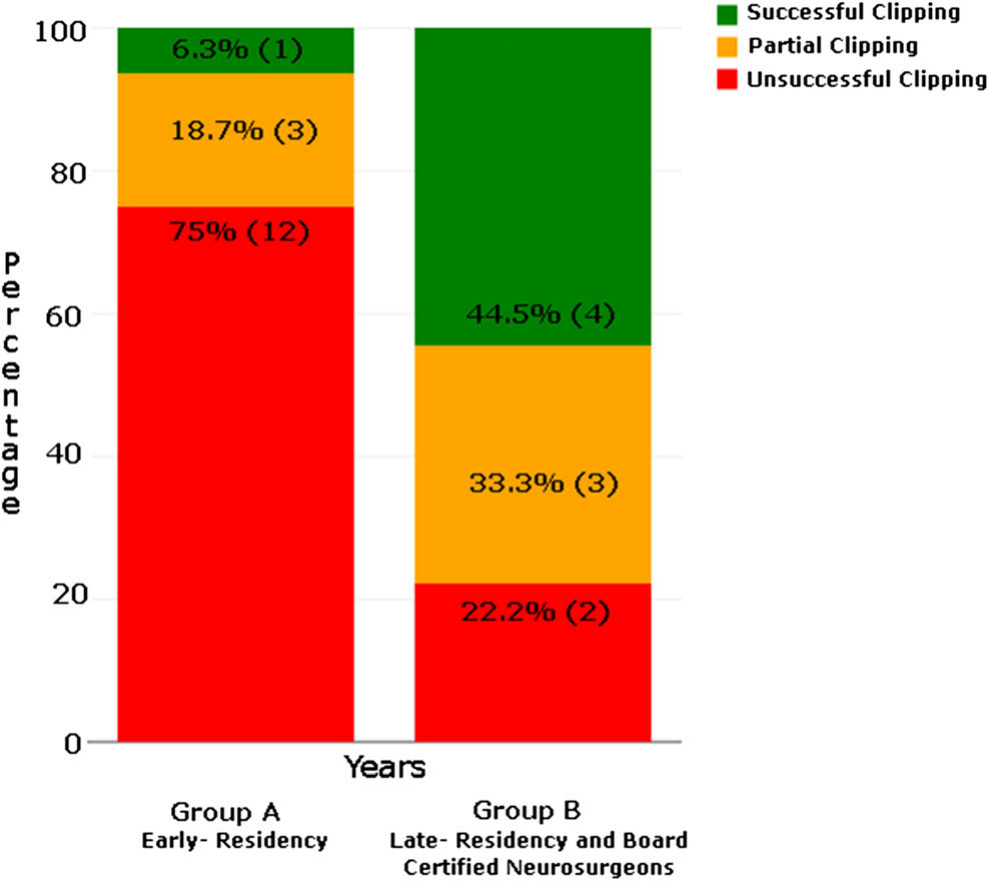
Nature of clipping (successful, unsuccessful, and partially done) vs. years of experience between group A and group B participants

A comparison of clipping performance between group A (early residency) and group B (late residency and board-certified neurosurgeons) is shown in Fig. 6. In group A, 12 out of 16 failed to clip, 3 partially clipped, and 1 clipped the aneurysm successfully. In group B, 2 out of 9 failed to clip, 3 partially closed the aneurysm, and 4 clipped successfully.

## Discussion

During surgery, neurosurgeons must be judgmental when choosing the matching clip(s) arrangement to completely occlude the intracranial aneurysm. Proven aneurysm remnant after clipping is reported in the literature to be as high as 14% [30], while surgical manipulation and aneurysm clipping put adjacent vessels at risk for stenosis, occlusion, or insult, this being potentially responsible for brain ischemia and infarction [31–34].

Cerebrovascular neurosurgery is and will always be motivated by technical skills, and the operating room is becoming an increasingly difficult environment for residents to obtain these skills. Residency work hour restrictions, the need to perform research, the increase in patient complexity, and the proliferation of alternative procedures have resulted in a reduction in hands-on operative learning opportunities and a change of required competencies. Several simulation training platforms are available for many medical disciplines and in a variety of forms like ex vivo models (cadaveric and animal), virtual reality handling, and static 3D anatomical demonstrators. Simulation training allows deliberate practice, progressive operative responsibility, and coaching by a senior surgeon. Simulators have been used to improve the performance of trainees in many areas of surgery and there is a growing demand for technological development in this area. Multiple studies have consistently demonstrated that the use of simulators improves qualitative and quantitative performance measures as well as overall resident and surgeon confidence in clinical settings. [35–38]

Although simulated training methods for practicing invasive procedures have been made available in other surgical specialties, realistic simulation options in cerebrovascular neurosurgery, including aneurysm clipping, are scanty. In neurosurgery, training modules reported so far have been missing blood-like flow time resolution (pulsatility) and other crucial phenomena like vascular patency, dynamic behavior, haptics, tactility, and the feedback of handling micro-surgical tools or to deal with intraoperative hemorrhage. This makes the surgeons unexposed to the complete surgical workflow. These aspects, together with the procedure-related morbidity, raise the need for functional, more realistic training simulators replicating the required surgical anatomy and physiology.

The recent development of additive manufacturing techniques leads us to develop a true scale, patient-specific bench-top simulator prototype.

The present study showed that more than 80% of the participants felt that our new anatomy simulation was a good alternative to theoretical and conventional learning [39]. All participants found the visual aspects of the simulation appealing, while most participants found the anatomical environment and the tactile and haptic aspects of the pathology in the simulation to be realistic. Participants’ responses to the multiple-choice questions indicated that over 80% were willing to use the simulator for education and training purposes. The same number felt that the simulator could be useful for patient-specific preoperative training in regular clinical practice and agreed that it would possibly improve their surgical skills.

The comparison of clipping performance between the groups showed, as intuitively expected, that trainees in their early stages of residency potentially need a long exposure to the simulator to improve their microsurgery clipping skills. In group B (late residency and board-certified neurosurgeons), 2 of the 9 participants also failed, perhaps because their exposure to this type of surgical procedure was limited, and perhaps because of the anatomy of the aneurysm we selected for this study was particularly challenging. This underlines the need for a validation study aiming to measure the learning curve of residents as they train with the simulator.

### Limitations and future directions

Despite our effort to reproduce the skull, brain, and vascular anatomy, further anatomic elements like the skin, galea, meninges, veins, and arachnoid were missing. This did not allow the complete surgical workflow to be reproduced.

Our study design and data did not allow us to compare the effectiveness of training. Future studies should focus on collecting long-term follow-up data and quantified parameters on simulated outcomes. This would allow us to measure the performance capacity and to establish a learning curve in order to determine whether there is an advantage to training in the simulated environment. To date, neurosurgeons have been using only one indicator of performance: patient outcomes. Our simulator could incorporate alternative measures to improve and maintain skills.

Further studies are needed in order to confirm that the present simulator is a valuable clinical tool for measuring, evaluating, and maintaining quality assurance in the training and education of future generations of neurosurgeons. These studies should include an assessment of the learning curve and skills improvement over time, with the goal of ensuring a high level of post-graduate education and patient care.

## Conclusion

The integration of a new surgical simulator including blood circulation and pulsatility should be considered as part of the future armamentarium of postgraduate education aimed to ensure high training standards for current and future generations of neurosurgeons involved in intracranial aneurysm surgery.

## References

[1] Belykh EG, Byval'tsev VA, Nakadzhi P, Lei T, Oliviero MM, Nikiforov SB (2014) [A model of the arterial aneurysm of the brain for microneurosurgical training]. Zh Vopr Neirokhir Im N N Burdenko 78(2):40–5– discussion 45 25033605

[2] Kang Y, Yu L-H, Xu T, Zheng S-F, Yao P-S, Liu M, Lin Y-X, Lin Z-Y, Fan X-M, Kang D-Z (2016). Three-dimensional printing technology for treatment of intracranial aneurysm. Chin Neurosurg Jl.

[3] Vakharia VN, Vakharia NN, Hill CS (2016). Review of 3-dimensional printing on cranial neurosurgery simulation training. World Neurosurg.

[4] Dewan MC, Rattani A, Fieggen G, Arraez MA, Servadei F, Boop FA, Johnson WD, Warf BC, Park KB (2018). Global neurosurgery: the current capacity and deficit in the provision of essential neurosurgical care. Executive Summary of the Global Neurosurgery Initiative at the Program in Global Surgery and Social Change. J Neurosurg.

[5] Kirkman MA, Ahmed M, Albert AF, Wilson MH, Nandi D, Sevdalis N (2014). The use of simulation in neurosurgical education and training. A systematic review. J Neurosurg.

[6] Lim PK, Stephenson GS, Keown TW, Byrne C, Lin CC, Marecek GS, Scolaro JA (2018). Use of 3D printed models in resident education for the classification of acetabulum fractures. Journal of Surgical Education.

[7] Williams A, McWilliam M, Ahlin J, Davidson J, Quantz MA, Bütter A (2018). A simulated training model for laparoscopic pyloromyotomy: Is 3D printing the way of the future?. J Pediatr Surg.

[8] Alaraj A, Luciano CJ, Bailey DP, Elsenousi A, Roitberg BZ, Bernardo A, Banerjee PP, Charbel FT (2015) Virtual reality cerebral aneurysm clipping simulation with real-time haptic feedback. Neurosurgery 1–7 10.1227/NEU.0000000000000583 PMC4340784 25599200

[9] Beier F, Sismanidis E, Stadie A, Schmieder K, Männer R (2012). An aneurysm clipping training module for the neurosurgical training simulator NeuroSim. Stud Health Technol Inform.

[10] Craven C, Baxter D, Cooke M, Carline L, Alberti SJMM, Beard J, Murphy M (2014). Development of a modelled anatomical replica for training young neurosurgeons. Br J Neurosurg.

[11] Koyama T, Hongo K, Tanaka Y, Kobayashi S (2000). Simulation of the surgical manipulation involved in clipping a basilar artery aneurysm: concepts of virtual clipping: Technical note. J Neurosurg.

[12] Kumagai K, Mori K, Takeuchi S, Wada K (2019). Surgical training for the management of intraoperative aneurysm rupture using a three-dimensional artificial model. Asian Journal of Neurosurgery.

[13] Benet A, Plata-Bello J, Abla AA, Acevedo-Bolton G, Saloner D, Lawton MT (2015). Implantation of 3D-printed patient-specific aneurysm models into cadaveric specimens: a new training paradigm to allow for improvements in cerebrovascular surgery and research. BioMed Research International.

[14] Waran V, Narayanan V, Karuppiah R, Pancharatnam D, Chandran H, Raman R, Rahman ZAA, Owen SLF, Aziz TZ (2014). Injecting realism in surgical training-initial simulation experience with custom 3D models. Journal of Surgical Education.

[15] Waran V, Narayanan V, Karuppiah R, Thambynayagam HC, Muthusamy KA, Rahman ZAA, Kirollos RW (2015). Neurosurgical endoscopic training via a realistic 3-dimensional model with pathology. Simul Healthc.

[16] Leal A, Souza M, Nohama P (2019). Additive manufacturing of 3D biomodels as adjuvant in intracranial aneurysm clipping. Artif Organs.

[17] Kimura T, Morita A, Nishimura K, Aiyama H, Itoh H, Fukaya S, Sora S, Ochiai C (2009) Simulation of and training for cerebral aneurysm clipping with 3-dimensional models. Neurosurgery 65(4):719–25– discussion 725–6 10.1227/01.NEU.0000354350.88899.07 19834377

[18] Liu Y, Ghassemi P, Depkon A, Iacono MI, Lin J, Mendoza G, Wang J, Tang Q, Chen Y, Pfefer TJ (2018). Biomimetic 3D-printed neurovascular phantoms for near-infrared fluorescence imaging. Biomed Opt Express.

[19] Raabe A, Nakaji P, Beck J, Kim LJ, Hsu FPK, Kamerman JD, Seifert V, Spetzler RF (2005). Prospective evaluation of surgical microscope-integrated intraoperative near-infrared indocyanine green videoangiography during aneurysm surgery. J Neurosurg.

[20] Mashiko T, Otani K, Kawano R, Konno T, Kaneko N, Ito Y, Watanabe E (2015). Development of three-dimensional hollow elastic model for cerebral aneurysm clipping simulation enabling rapid and low cost prototyping. WNEU.

[21] Wurm G, Lehner M, Tomancok B, Kleiser R, Nussbaumer K (2011). Cerebrovascular biomodeling for aneurysm surgery: simulation-based training by means of rapid prototyping technologies. Surg Innov.

[22] Kaneko N, Mashiko T, Namba K, Tateshima S, Watanabe E, Kawai K (2018). A patient-specific intracranial aneurysm model with endothelial lining: a novel in vitro approach to bridge the gap between biology and flow dynamics. J NeuroIntervent Surg.

[23] Scerrati A, Trovalusci F, Albanese A, Ponticelli GS, Tagliaferri V, Sturiale CL, Cavallo MA, Marchese E (2019). A workflow to generate physical 3D models of cerebral aneurysms applying open source freeware for CAD modeling and 3D printing. Interdisciplinary Neurosurgery.

[24] Joseph FJ, Bervini D, Raabe A, Weber S (2019) Production of intracranial dynamic aneurysm models for neurosurgical applications and future directions. Joseph, Fredrick Johnson; Bervini, David; Raabe, Andreas; Weber, Stefan (19 September 2019) Production of intracranial dynamic aneurysm models for neurosurgical applications and future directions In: 18 Jahrestagung der Deutschen Gesellschaft für Computer- und Roboterassistierte Chirurgie (pp 108-111) CURAC 2019. doi: 10.7892/boris.134185

[25] Kaneko N, Mashiko T, Ohnishi T, Ohta M, Namba K, Watanabe E, Kawai K (2016). Manufacture of patient-specific vascular replicas for endovascular simulation using fast, low-cost method. Scientific Reports 2018 8:1.

[26] Ryan JR, Almefty KK, Nakaji P, Frakes DH (2016). Cerebral aneurysm clipping surgery simulation using patient-specific 3D printing and silicone casting. World Neurosurg.

[27] Luo M, Frisken SF, Weis JA, Clements LW, Unadkat P, Thompson RC, Golby AJ, Miga MI (2017). Retrospective study comparing model-based deformation correction to intraoperative magnetic resonance imaging for image-guided neurosurgery. J Med Imaging (Bellingham).

[28] Harris PA, Taylor R, Minor BL (2019). The REDCap consortium: building an international community of software platform partners. Journal of Biomedical Informatics.

[29] Harris PA, Taylor R, Thielke R, Payne J, Gonzalez N, Conde JG (2009). Research electronic data capture (REDCap)—a metadata-driven methodology and workflow process for providing translational research informatics support. Journal of Biomedical Informatics.

[30] Sindou M, Acevedo JC, Turjman F (1998). Aneurysmal remnants after microsurgical clipping: classification and results from a prospective angiographic study (in a consecutive series of 305 operated intracranial aneurysms). Acta Neurochir (Wien).

[31] Bekelis K, Missios S, MacKenzie TA, Desai A, Fischer A, Labropoulos N, Roberts DW (2014). Predicting inpatient complications from cerebral aneurysm clipping: the Nationwide Inpatient Sample 2005–2009. J Neurosurg.

[32] Bruneau M, Amin-Hanjani S, Koroknay-Pal P (2016). Surgical clipping of very small unruptured intracranial aneurysms. Neurosurgery.

[33] Bulters DO, Santarius T, Chia HL, Parker RA, Trivedi R, Kirkpatrick PJ, Kirollos RW (2010). Causes of neurological deficits following clipping of 200 consecutive ruptured aneurysms in patients with good-grade aneurysmal subarachnoid haemorrhage. Acta Neurochir (Wien).

[34] Le Roux PD, Elliott JP, Eskridge JM, Cohen W, Winn HR (1998). Risks and benefits of diagnostic angiography after aneurysm surgery: a retrospective analysis of 597 studies. Neurosurgery.

[35] Clark AD, Barone DG, Candy N, Guilfoyle M, Budohoski K, Hofmann R, Santarius T, Kirollos R, Trivedi RA (2017). The effect of 3-dimensional simulation on neurosurgical skill acquisition and surgical performance_ a review of the literature. Journal of Surgical Education.

[36] Flemming B, Skou TAS, Joy NL, Lars K (2020). Surgical simulation: current practices and future perspectives for technical skills training. Medical Teacher.

[37] Johnston MJ, Paige JT, Aggarwal R, Stefanidis D, Tsuda S, Khajuria A, Arora S (2016). An overview of research priorities in surgical simulation: what the literature shows has been achieved during the 21st century and what remains. The American Journal of Surgery.

[38] Yanagawa B, Ribeiro R, Naqib F, Fann J, Verma S, Puskas JD (2019). See one, simulate many, do one, teach one. Current Opinion in Cardiology.

[39] de Oliveira MMR, Ferrarez CE, Ramos TM (2018). Learning brain aneurysm microsurgical skills in a human placenta model: predictive validity. J Neurosurg.

